# Epidemiology of ocular surface squamous neoplasia in veterans: a retrospective case-control study

**DOI:** 10.1186/s40662-019-0138-1

**Published:** 2019-05-14

**Authors:** Logan M. Smith, Shiv Lamba, Carol L. Karp, Anat Galor

**Affiliations:** 1 Miami Veterans Administration Medical Center, 1201 NW 16th St, Miami, FL 33125 USA; 2 Bascom Palmer Eye Institute, Department of Ophthalmology, University of Miami, 900 NW 17th Street, Miami, FL 33136 USA; 3 Flint Hill School, Oakton, Virginia USA

**Keywords:** Ocular surface squamous neoplasia, Risk factors, Veteran population

## Abstract

**Background:**

A number of risk factors have been evaluated in ocular surface squamous neoplasia, but few studies have assessed risk factors specific to the armed forces veteran population.

**Methods:**

We conducted a retrospective case-control study on 55 patients and 55 age-matched controls with biopsy-proven ocular surface squamous neoplasia from the Miami Veterans Administration Hospital Eye Clinic to investigate potential risk factors encountered by veterans, including service-specific exposures. Veteran-specific risk factors included ionizing radiation exposure, Agent Orange exposure, deployment to Southwest Asia, and exposure to the series of biochemical warfare tests known as Project Shipboard Hazard and Defense. Data was analyzed with SPSS (SPSS Inc., Chicago, IL) using t-tests, chi-squared, and logistic regression analysis, with a *p*-value of < 0.05 considered statistically significant.

**Results:**

The strongest risk factor for ocular surface squamous neoplasia was lifetime sun exposure both directly assessed via historical quantification of exposure by dermatology practitioners (Odds Ratio (OR) 5.4, 95% Confidence Interval (CI) 2.27–12.847, *p* < 0.005), and using the surrogate markers of basal cell carcinoma (OR 3.157, 95% CI 1.286–7.748, *p* = 0.010) and pingueculae (OR 5.267, 95% CI 2.104–13.186, *p* < 0.005). Of the veteran-specific risk factors, Agent Orange exposure and Southwest Asia deployment were not associated with an increased risk of ocular surface squamous neoplasia. Exposure to ionizing radiation and involvement in Project Shipboard Hazard and Defense were not documented among any cases or controls.

**Conclusions:**

The results of our study are consistent with prior established risk factors, namely highlighting the important role of sun exposure in ocular surface squamous neoplasia among veterans.

## Background

Ocular surface squamous neoplasia (OSSN) represents a range of pathologies of the ocular surface ranging from squamous epithelial dysplasia to invasive squamous cell carcinoma (SCC) arising from the conjunctiva, cornea or both [1]. While large-scale epidemiological studies are lacking as a whole, a study carried out by the United States National Cancer Institute estimated an annual incidence of 0.03/100,000 for SCC arising from the conjunctiva of Caucasian patients [2].

To increase understanding of this relatively uncommon disease, risk factors for the development of OSSN have been researched. Excess ultraviolet (UV) radiation and its sequelae in the eye (pingueculae, pterygia, etc.) remains one of the most thoroughly associated exposures [3]. Other factors associated with OSSN include human immunodeficiency virus (HIV) [4], human papillomavirus (HPV) [5, 6], allergic conjunctivitis [7], and genetic mutations involving impaired DNA repair, such as xeroderma pigmentosum and defects in the tumor suppressor gene *TP53* which plays an important role in regulation of the cell cycle [8]. The association between smoking and OSSN has also been researched with mixed results [9, 10].

Two distinct clinical presentations of OSSN have been described in the literature. In developed countries such as the United States and Australia, OSSN tends to be more prevalent amongst elderly white men in their sixties [11]. In contrast, cases of OSSN in developing nations like sub-Saharan Africa tend to be diagnosed in young black patients (male and female) in their mid-to-late 30’s [12, 13]. Patients diagnosed in developing countries also tend to present with much more aggressive disease, which has been hypothesized to be due to the higher prevalence of HIV and HPV [14, 15].

As OSSN typically presents amongst elderly white males in developed countries, a particular population of interest is the aging veteran population. Members of the armed forces are exposed to unique factors during their terms of deployment, and limited research has been done to examine the impact of these exposures on the development of OSSN. A study by McClellan et al. performed in 2015 investigated risk factors for 28 cases of OSSN who presented for evaluation to the Veterans Administration Hospital of Miami from 2007 to 2012, showing that factors associated with UV light exposure predominated [16]. However, this study did not research the impact of veteran-specific risk factors, such as exposure to ionizing radiation, Agent Orange, deployment to Southwest Asia, and involvement in a series of biochemical warfare tests conducted from 1962 to 1973 known as Project Shipboard Hazard and Defense [17], on OSSN. With this in mind, our study aimed to assess the impact of veteran-specific risk factors on the development of OSSN.

## Methods

### Patient population

The study population included 55 cases of biopsy-proven OSSN who were treated at the Miami Veterans Administration Hospital Eye Clinic between 2010 and 2018. Fifty-five age- (within 5 years), gender- and race-matched controls without OSSN were selected from individuals seen in the Miami Veterans Administration Hospital Eye Clinic on the same dates as the cases.

### Data collection

The Miami Veterans Administration Institutional Review Board approved this retrospective case-control study. The methods adhered to the tenets of the Declaration of Helsinki and were Health Insurance Portability and Accountability Act (HIPAA)-compliant. Data were acquired from the Veterans Administration Hospital electronic medical record system. Demographic information included age, gender and race. Past medical history included diagnoses of type 2 diabetes mellitus, head and neck cancer and xeroderma pigmentosum. General risk factors investigated included tobacco exposure (current or previous vs. none), infection with HPV or infection with HIV. Ultraviolet-related exposures included history of significant lifetime sun exposure (coded as yes or no based on dermatology note documentation); history of outdoor occupation outside of military service (coded as yes or no; primary care notes documented occupations, those we considered outdoor were construction workers, fishermen, boat workers, gardeners and farmers); skin pathologies (actinic keratosis, basal cell carcinoma (BCC), SCC, melanoma); and diagnoses of pingueculae or pterygia. Veteran-specific risk factors were also extracted, including exposure to ionizing radiation, Agent Orange, Southwest Asia deployment [18]; and involvement in Project Shipboard Hazard and Defense (all coded as yes or no based on documentations in the medical record).

### Statistical analysis

Descriptive statistics were used to summarize our patient population. Statistical analyses were performed using Microsoft Excel Version 16.12 and SPSS Version 25 (SPSS Inc., Chicago, IL). Analysis was performed on 55 biopsy-proven cases of OSSN and their corresponding controls. Continuous variables were compared between cases and controls using an independent t-test and categorical variables were compared using Chi squared or Fisher exact tests, as appropriate. Logistic regression analysis was used to evaluate the effect of risk factors on OSSN, as represented by an odds ratio (OR). A 95% confidence interval (CI) was determined with a *p*-value < 0.05 being considered statistically significant.

## Results

### Demographics

The mean age of the cases on chart review was 73.2 ± 8.7 years with OSSN being diagnosed at a mean age of 69.1 ± 9.2 years. The mean age of the control population was 72.9 ± 8.0 years. Nearly all patients were male (98%) and Caucasian (85.5%). No statistically significant association with OSSN was found in relation to age, gender or race (Table 1).

### Medical conditions

Among the medical factors examined, no significant association with OSSN was found for type 2 diabetes mellitus, head/neck cancer or xeroderma pigmentosum (Table 1).

### General risk factors

No significant association with OSSN was found for general risk factors including history of current or former tobacco exposure, infection with HPV or infection with HIV.

### UV-related risk factors

Among UV-related risk factors, a history of significant sun exposure (as documented by dermatology) (OR 5.40, 95% CI 2.27–12.85, *p* = < 0.005), history of outdoor occupation as a boat worker (OR 1.08, 95% CI 1.01–1.16, *p* = 0.04) as well as diagnoses of BCC (OR 3.16, 95% CI 1.29–7.75, *p* = 0.01) and pingueculae (OR 5.27, 95% CI 2.10–13.19, *p* = < 0.005) were all significantly associated with OSSN. History of any of the outdoor occupations investigated (OR 1.92, 95% CI 0.76–4.86, *p* = 0.17) as well as diagnoses of actinic keratosis (OR 1.24, 95% CI 0.59–2.63, *p* = 0.57), melanoma (OR 4.24, 95% CI 0.46–39.2, *p* = 0.17) and pterygia (OR 2.29, 95% CI 0.92–5.71, *p* = 0.07) also exhibited elevated risk of OSSN but did not reach statistical significance (Table 1).

### Veteran-specific risk factors

Among the veteran-specific risk factors studied, none significantly affected the risk of OSSN. Agent Orange exposure (OR 1.37, *p* = 0.5) had an OR greater than 1 but was not statistically significant. Exposure to ionizing radiation and involvement in Project Shipboard Hazard and Defense were not documented among any cases or controls, and Southwest Asia condition was only documented in a handful of controls and no cases (Table 1).

**Table 1:** Demographics and risk factors for ocular surface squamous neoplasia (OSSN) and control populations

Demographics	OSSN (*n* = 55)	Controls (*n* = 55)	OR (95% CI)	*P*-value
Age, mean (SD) [range] years	73.2 (8.7) [40–98]	72.9 (8.0) [45–97]	1.00 (0.96–1.05)	0.87
Gender, male, n%	54 (98.2%)	54 (98.2%)	1.00 (0.06–16.40)	1.00
Race			1.00 (0.35–2.89)	1.00
White, n%	47 (85.4%)	47 (85.4%)		
Black, n%	8 (14.6%)	8 (14.6%)		
*Past Medical History*				
Type 2 diabetes mellitus, n%	17 (30.9%)	20 (36.4%)	0.92 (0.41–0.06)	0.84
Head/neck cancer, n%	4 (7.3%)	4 (7.3%)	1.00 (0.24–4.220)	1.00
Xeroderma pigmentosum, n%	0 (0%)	0 (0%)	N/A*	N/A*
*General Risk Factors*				
Tobacco, current or previous, n%	40 (72.7%)	35 (63.6%)	1.19 (0.53–2.67)	0.68
HPV, n%	4 (7.3%)	2 (3.6%)	2.08 (0.37–11.9)	0.41
HIV, n%	1 (1.8%)	1 (1.8%)	1.00 (0.06–16.4)	1.00
*UV-related Exposures*				
Significant sun exposure, n%	30 (54.5%)	10 (18.2%)	5.40 (2.27–12.9)	< 0.005
History of outdoor occupation, n%	15 (27.3%)	9 (16.4%)	1.92 (0.76–4.86)	0.17
Construction worker	7	8	0.86 (0.29-2.55)	0.78
Fisherman	1	1	1.00 (0.61–16.40)	1.00
Boat worker	4	0	1.08 (1.01–1.16)	0.04
Gardener	2	0	1.04 (0.99–1.09)	0.15
Farmer	1	0	1.02 (0.98–1.06)	0.32
Actinic keratosis, n%	28 (50.9%)	26 (47.3%)	1.24 (0.59–2.630)	0.57
BCC, n%	21 (38.1%)	9 (16.4%)	3.16 (1.29–7.75)	0.01
SCC, n%	9 (16.4%)	13 (23.6%)	0.70 (0.27–1.83)	0.47
Melanoma, n%	4 (7.3%)	1 (1.8%)	4.24 (0.46–39.2)	0.17
Pinguecula, n%	26 (47.3%)	8 (14.5%)	5.27 (2.10–13.19)	< 0.005
Pterygium, n%	17 (30.9%)	9 (16.4%)	2.29 (0.92–5.71)	0.07
*Veteran-specific exposures*				
Ionizing radiation, n%	0 (0%)	0 (0%)	N/A*	N/A*
Agent Orange, n%	14 (25.4%)	11 (20%)	1.37 (0.56–3.35)	0.50
Southwest Asia deployment, n%	0 (0%)	3 (5.5%)	0.49 (0.40–0.59)	0.08
Involvement in Project Shipboard Hazard and Defense, n%	0 (0%)	0 (0%)	N/A*	N/A*

*SD=* standard deviation, *n=* number in each group, *BCC=* Basal cell carcinoma, *SCC=* Squamous Cell Carcinoma, *HPV=* Human Papilloma Virus, *HIV=* Human Immunodeficiency Virus, *OR=* Odds Ratio, *CI=* Confidence Interval, *N/A=* not applicable

*P-values unable to be calculated

## Discussion

While the study by McClellan et al. investigated well-researched risk factors for OSSN in the veteran population of South Florida [16], our study sought to continue this work by investigating the effect of veteran-specific factors on OSSN. Of these veteran-specific risk factors, no statistically significant associations with OSSN were found. On the other hand, our results confirm that factors associated with increased UV exposure (history of significant lifetime sun exposure, occupation as a boat worker, BCC and pinguecula) were significantly associated with an increased risk of OSSN. Other factors associated with increased UV exposure (history of any outdoor occupation, actinic keratosis, melanoma, pterygia) also exhibited increased risk of OSSN but did not reach statistical significance. Similar to the study by McClellan, we showed a paradoxically decreased risk of developing OSSN with a history of type 2 diabetes mellitus but our association did not reveal any statistical significance.

UV exposure has a strong association with the development of OSSN and our findings supported this [3]. Longer wavelengths of UV radiation (particularly UV-B) are absorbed by DNA strands leading to the production of cyclobutene pyrimidine dimers via the formation of carbon-carbon double bonds in the nucleotide’s associated nitrogenous bases [19]. These pyrimidine dimers have been shown to inhibit the function of DNA polymerases, and failure of cells to repair UV-induced damage to DNA can lead to misreading of the genetic code, leading to mutations and neoplasia [20]. Other DNA-damaging reactions that occur as a result of UV exposure include hydrolytic deamination of bases (which in turn alter the genetic code at that segment) and the generation of reactive oxygen species [21]. The skin and eyes are both superficial structures and have the greatest exposure to UV, leading a spectrum of benign (thinning of the skin, cataracts) and neoplastic (SCC, melanoma) conditions [22, 23].

Many veterans are exposed to UV radiation. Unique exposures for veterans include service in regions and armed forces branches with increased sun exposure, such as aboard waterboard vessels. A survey of active service men and women showed that less than 30% of soldiers had regular access to sunscreen and that as a result their skin was unprotected 70% or more of the time [24]. Large scale systematic reviews have shown that US military personnel have higher rates of skin cancer than civilians along with lower rates of skin cancer awareness and dermatologic care [25]. In addition to service, occupational exposure while not deployed represents another route for UV irradiation. In our population, 22% of individuals had a career that required outdoor sun exposure with those serving as boat workers having a significantly higher risk of developing OSSN.

We additionally focused on other veteran-specific risk factors because there is biological plausibility that some of these may increase the risk of eye malignancy. Exposure to ionizing radiation has long been shown to be carcinogenic. After the discovery of X-rays in the late nineteenth century, the development of SCC in exposed skin was recognized by early radiologists [26]. Some members of the armed forces, such as nuclear weapon technicians, have increased exposure to ionizing radiation, increasing their risk for malignancy. A case study by Nelson et al. details a veteran patient exposed to radioactive debris during salvage of material from a failed atmospheric weapon launch who subsequently went on to develop over 300 BCC’s and 2 SCC’s over a 30-year period, hypothesized to be due to ionizing radiation exposure from the atomic weapon [27]. In addition to skin cancers, a large scale study performed in Australia showed that exposure to low-dose ionizing radiation via computed tomography in childhood led to an increased incidence of a variety of cancers later in life including brain, thyroid, urinary tract, genital organs, and hematologic malignancies [28]. However, none of the individuals in our cohort had documentation of exposure to ionizing radiation.

Agent Orange’s role as a risk factor for malignancy has also been researched extensively. A contaminant in the production of Agent Orange, 2,3,7,8-tetrachlorodibenzo-*p* dioxin (TCDD), is a known human carcinogen [29]. The proposed mechanism of TCDD involves stimulation of the aryl hydrocarbon receptor (AhR), which upregulates cellular signaling, DNA binding and transcriptional activation leading to tumor-promoting activities [30]. Evidence for this mechanism has been strengthened by studies showing that AhR-deficient mice show resistance to TCDD-induced toxicity [31]. Beginning in 1994, the Institute of Medicine has issued periodic reports on Agent Oranges effects on both cancerous and non-cancerous health conditions. As of the latest report, exposure to Agent Orange has shown sufficient evidence of association to the development of soft tissue sarcomas, Non-Hodgkin’s and Hodgkin’s lymphoma and chronic lymphocytic lymphoma in the veteran population [32]. While some studies suggested that TCDD could affect squamous cells as well [33, 34], exposure to Agent Orange was not found to be a significant risk factor for OSSN in our study.

Involvement in Project Shipboard Hazard and Defense represents another avenue that veterans could be exposed to possibly carcinogenic chemicals. In the 1960’s, the U.S. Department of Defense conducted a series of tests to investigate vulnerabilities of U.S. Navy ships to chemical and biological agents, such as the nerve gases sarin and venomous agent X (commonly referred to as “VX”). It was believed that approximately 5900 veterans were involved in the testing. Long-term follow up studies on mortality in veterans involved in Project Shipboard Hazard and Defense showed no increased mortality associated with malignancies but did demonstrate an increased overall mortality due to heart disease [35]. Since none of our patients took part in Project Shipboard Hazard and Defense, we were unable to evaluate if there is any association with OSSN.

A number of limitations were present within our study that must be considered when interpreting the study results. Our data is limited to that available within the VA electronic medical records. OSSN has a relatively low incidence and our study population was limited to fifty five cases of OSSN over eight years of review. Exposure to veteran-specific risk factors of interest, such as ionizing radiation and Project Shipboard Hazard and Defense, was not found during chart review, limiting our ability to assess their role in OSSN. Future studies specifically including patients exposed to ionizing radiation and Project Shipboard Hazard and Defense can better delineate if these exposures are significant risk factors for OSSN development. Collaboration between multiple institutions could also increase the size of the study population and power of future studies. We also did not assess the effect of newly appreciated veteran exposures such as uranium or petroleum as these information were not readily available within the medical records.

## Conclusions

Despite the limitations discussed, our study corroborates the impact UV light has on OSSN. Further research into risk factors in specific populations will help identify new risk factors and allow for evidence-guided screening protocols in appropriate populations.

## Abbreviations

BCC: Basal cell carcinoma
CI: Confidence interval
HIV: Human immunodeficiency virus
HPV: Human papilloma virus
OR: Odds ratio
OSSN: Ocular surface squamous neoplasia
SCC: Squamous cell carcinoma
TCDD: tetrachlorodibenzo-*p* dioxin
UV: Ultraviolet
